# All-Dielectric Metasurface-Based Terahertz Molecular Fingerprint Sensor for Trace Cinnamoylglycine Detection

**DOI:** 10.3390/bios14090440

**Published:** 2024-09-13

**Authors:** Qiyuan Xu, Mingjun Sun, Weijin Wang, Yanpeng Shi

**Affiliations:** School of Integrated Circuits, Shandong University, Jinan 250100, China

**Keywords:** terahertz, molecular fingerprint sensor, all-dielectric metasurface, cinnamoylglycine, gestational diabetes mellitus

## Abstract

Terahertz (THZ) spectroscopy has emerged as a superior label-free sensing technology in the detection, identification, and quantification of biomolecules in various biological samples. However, the limitations in identification and discrimination sensitivity of current methods impede the wider adoption of this technology. In this article, a meticulously designed metasurface is proposed for molecular fingerprint enhancement, consisting of a periodic array of lithium tantalate triangular prism tetramers arranged in a square quartz lattice. The physical mechanism is explained by the finite-difference time-domain (FDTD) method. The metasurface achieves a high quality factor (Q-factor) of 231 and demonstrates excellent THz sensing capabilities with a figure of merit (FoM) of 609. By varying the incident angle of the THz wave, the molecular fingerprint signal is strengthened, enabling the highly sensitive detection of trace amounts of analyte. Consequently, cinnamoylglycine can be detected with a sensitivity limit as low as $1.23\;\mu g\cdot {cm}^{-2}$. This study offers critical insights into the advanced application of THz waves in biomedicine, particularly for the detection of urinary biomarkers in various diseases, including gestational diabetes mellitus (GDM).

## 1. Introduction

Terahertz (THz) spectroscopy, a portion of the electromagnetic spectrum, deals with radiation frequencies from 0.1 THz to 10 THz. This radiation can penetrate a wide variety of non-conducting materials and exhibits non-ionizing properties [1]. Consequently, it is acclaimed as a potent spectroscopic technique due to its label-free and non-destructive attributes. Previous research in THz time-domain spectroscopy has demonstrated it to be a state-of-the-art technology capable of providing distinct molecular fingerprints by detecting vibrational and rotational modes sensitive to molecular structure and environmental factors within the THz frequency range [2,3]. This underscores its substantial potential for applications in biological detection [3,4]. However, traditional THz spectroscopy encounters limitations, notably its reduced sensitivity for detecting trace analytes, which impedes its extensive application. This issue arises because the dimensions of target molecules are significantly smaller than the micrometer scale, whereas the wavelength of THz waves spans approximately from 30 μm to 3000 μm [5], leading to a remarkable mismatch between the absorption cross-section of the analyte and the wavelength. Hence, the interactions between them are too feeble to generate adequate vibrational absorption signals, necessitating a substantial quantity of analyte [6,7]. Recent studies have proposed various approaches to address this issue, encompassing surface plasmon resonances (SPRs) [8], electromagnetic subwavelength structures [9], waveguides [10], metal hole arrays [11], and graphene metamaterials [12]. These sensors can achieve sub-wavelength spatial resolution to enhance detection sensitivity [5]. But they are constrained by substantial ohmic losses and high dispersion of the metal materials [13,14], leading to limited improvements in sensitivity [15,16].

All-dielectric metasurface has been demonstrated as an alternative to traditional metallic metasurfaces. It is based on high-refractive-index dielectrics and leverages Mie-type resonances rather than surface plasmon resonances to obtain strong magnetic resonances as well as electric resonances, thereby enhancing the interaction between electromagnetic waves and materials [17,18,19]. This innovative design significantly reduces energy losses while elevating the quality factor (Q-factor) of the resonators, thus greatly improving detection sensitivity [17,20]. Furthermore, intrinsic heating can alter the local refractive index and potentially damage biomolecules. A dielectric metasurface can mitigate the plasmonic ohmic loss and heating issues, thereby enabling better repeatability and stability for biosensing [21]. Currently, a precise angle-scanning strategy has been proposed, which is based on guided-mode resonance theory [22]. By monitoring the transmission characteristics of THz waves at different incident angles, richer spectral information can be obtained, thereby improving the detection capability of trace analytes [22,23]. Employing this strategy, a previous study successfully identified lactose and glucose with detection limits of $1.53\;\mu g\cdot {cm}^{-2}$ and $1.54\;\mu g\cdot {cm}^{-2}$, respectively [24]. On this basis, maintaining the polarization direction can further refine the enhancement effect and broaden the bandwidth [24].

Cinnamoylglycine, a metabolic byproduct of cinnamic acid metabolism, is derived from dietary sources such as cinnamon [25]. In the human body, it is produced by gut microbes [26] and excreted in urine [27]. Gestational diabetes mellitus (GDM), which involves significant metabolic alterations [28,29], can pose severe health risks to both mother and fetus, such as preeclampsia [30], preterm birth [31], and increased risk of type-2 diabetes later in life [32]. These harms highlight the need for efficient GDM diagnostic methods for regular monitoring and timely intervention. Current methods, including fasting plasma glucose [33,34], oral glucose tolerance testing [35], and the detection of glycated hemoglobin [36], have limitations such as the need for fasting, multiple blood draws, and variability due to external factors. In contrast, studies have suggested a link between urinary cinnamoylglycine levels and diabetes, with diabetic patients exhibiting a clearance rate of 77 mL/min compared to 317 mL/min in healthy controls [37,38,39]. This proves urinary cinnamoylglycine levels are a practicable biomarker for monitoring metabolic changes associated with GDM and offer a non-invasive alternative. But existing detection methods for cinnamoylglycine, such as high-performance liquid chromatography (HPLC), are time-consuming and costly due to the pretreatment and processing of such highly complex matrices [40]. This underscores the critical need for a rapid, convenient, and accurate detection method for urinary cinnamoylglycine, thereby enhancing the overall management and prognosis of GDM. THz spectroscopy has proven effective for analyzing urine samples and biomarkers [41]. Furthermore, the use of metamaterials has created additional opportunities over the past decade, such as the detection of proteins [42,43] and cancer biomarkers [44]. Therefore, this study introduces a THz sensor to urinary cinnamoylglycine detection, utilizing a sophisticatedly designed all-dielectric metasurface to enhance molecular fingerprints and improve detection sensitivity and specificity.

In this study, we propose an all-dielectric metasurface-based THz sensor, utilizing a periodic symmetric tetramer of lithium tantalate triangular prisms on a quartz substrate. This periodic unit structure, composed of the four high-index tetramer clusters, can induce a strong magnetic dipole (MD) resonance, with a high Q-factor of 231 and figure of merit (FoM) of 609, thus significantly enhancing the molecular fingerprint for detecting trace amounts of analytes. By manipulating the incident angle of a THz wave, a broad envelope curve can be measured due to the shift of resonance frequency, which corresponds to the absorption resonances of the analyte. Consequently, this innovative sensor allows for the detection of cinnamoylglycine, with a detection limit as low as $1.23\;\mu g\cdot {cm}^{-2}$. Moreover, the proposed metasurface can be efficiently fabricated using micro/nanotechnology, and the testing can be performed with a terahertz time-domain spectrometer [45].

## 2. Structure and Design

**Figure 1:**
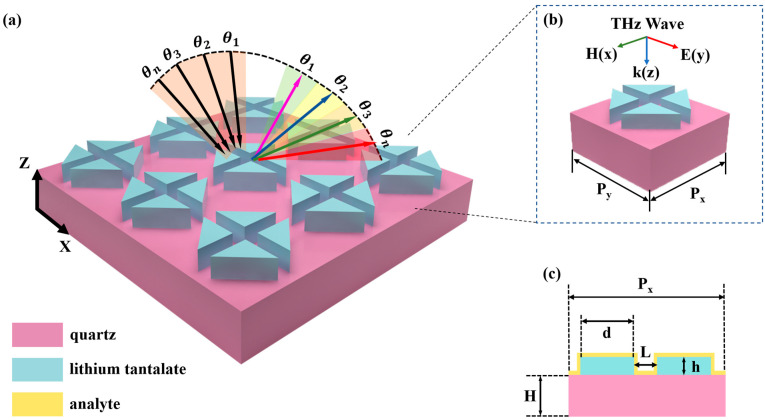
(**a**) The structural diagram of the all-dielectric metasurface, illustrating the periodic arrangement of the high-index triangular prism tetramer based on the quartz substrate; (**b**) a unit cell of the periodic structure with a y-polarized source incident downwards in the z direction; (**c**) the main view of the unit cell (y–z plane) and corresponding parameters.

Figure 1 depicts the schematic of the proposed all-dielectric metasurface. It consists of a periodic array of tetramer clusters arranged on a square quartz substrate. Each tetramer cluster is formed by four high-index triangular prisms. The dielectric property is a crucial characteristic of a material, as it fundamentally defines how the material responds to electromagnetic waves. It uniquely determines how radiation propagates through the material by describing its ability to polarize in response to an electric field, thus influencing the propagation of waves within the medium [46]. Lithium tantalate is chosen as the building material for the tetramer clusters due to its minimal imaginary part, resulting in negligible dissipation loss within the relevant frequency range [47]. The complex dielectric permittivity of lithium tantalate can be described as a Lorentz-type dispersion, as is described by the following equation [48]:

(1) $$\varepsilon =\varepsilon_{\infty }\frac{\omega^{2}-{\omega_{L}}^{2}+i\omega \gamma }{\omega^{2}-{\omega_{T}}^{2}+i\omega \gamma }\;,$$

In the equation, the transverse and longitudinal optical phonon frequencies are $\omega_{T}/2\pi =26.7\;THz$ and $\omega_{L}/2\pi =46.9\;THz$, respectively. The damping factor is γ/2=0.94 THz and the quartz substrate has a refractive index of n=2. For frequencies below the longitudinal optical phonon frequency, the permittivity of lithium tantalate is calculated to be 41.4 [49].

The geometric parameters of the square unit cell are illustrated in Figure 1b,c, with the side length $P_{x}=P_{y}=135\;\mu m$ and the substrate thickness H=130 μm. The prisms are shaped as isosceles right-angled triangles, of which the side length is d=90.4 μm. As shown in the main view of the unit cell in Figure 1c, the distance between adjacent triangular prisms is L=33 μm, and the height of each prism is h=49.6 μm. By directing the incident THz wave to propagate downward along the z direction, the resonance dip within the transmission spectrum can be manipulated, resulting in the formation of an envelope curve that captures the absorption resonances of analytes. This dynamic shifting of the resonance dip is essential for identifying the molecular fingerprints of various substances.

In order to evaluate the performance of the proposed sensor, extensive numerical simulations and spectral response analysis have been carried out with the commercially available three-dimensional finite-difference time-domain (FDTD) software. In the simulation, periodic boundary conditions were applied along the x and y directions to emulate an infinite array, while a perfectly matched layer boundary condition was set along the z-axis to prevent wave reflections. The simulations were conducted with a minimum meshing step of 1 nm to achieve high-resolution accuracy in modeling the electromagnetic fields.

## 3. Results and Discussion

**Figure 2:**
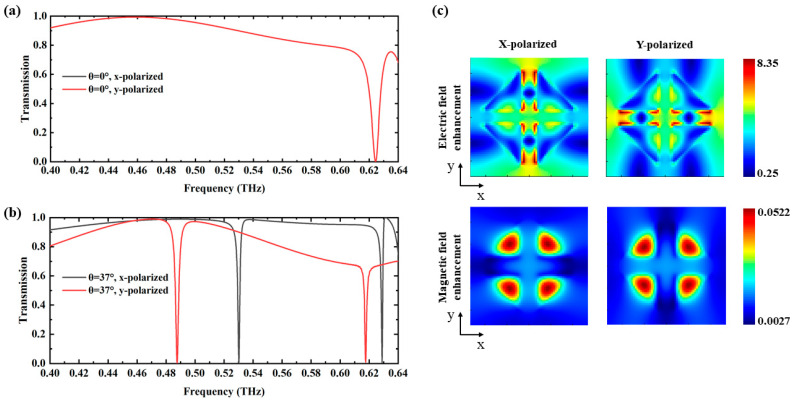
(**a**) Transmission spectra for x-polarized and y-polarized incident waves at 0°; (**b**) transmission spectra for x-polarized and y-polarized incident waves at 37°; (**c**) the electric and magnetic field distribution measured at the surface of the quartz substrate at vertical incidence. The left and right figures correspond to the x-polarized and y-polarized incident wave, respectively.

Figure 2 illustrates the transmission spectra of the proposed metasurface and the electric and magnetic field at the resonance frequency. In Figure 2a, a single resonance at 0.624 THz is observed for both x-polarized and y-polarized waves under normal incidence due to the symmetric arrangement of the metasurface structure. However, as shown in Figure 2b, when the incident angle is adjusted to 37°, the sensor exhibits a strong sensitivity to polarization due to the breaking in symmetry, leading to a frequency shift. The resonance is measured at 0.487 THz for y-polarized waves, showing strong correspondence with the absorption peak of cinnamoylglycine and a high Q-factor of approximately 231. The Q-factor is defined as $Q=f_{0}/∆f$, where the full-width at half-maximum (FWHM) ∆f is 2.11 GHz and the resonance frequency $f_{0}$ is 0.487 THz. In contrast, the frequency shift for x-polarized waves does not align sufficiently with the targeted absorption peak. Therefore, y-polarized waves were selected for angle-scanning to achieve frequency shifts and obtain the transmission envelope curve.

Figure 2c shows the electric and magnetic field distributions at the substrate surface in the x–y plane. The left and right panels correspond to x-polarized and y-polarized incident waves, respectively. The resonance is identified as an MD mode, characterized by the collective response of four longitudinal MDs [50]. The electric field is predominantly localized in the center region of the cluster, indicating the excitation of the MD resonance in this area, which enhances the interaction between the incident THz waves and analytes. The magnetic field distribution confirms the presence of strong MD resonances, further supporting the sensor’s sensitivity to different polarization states and incident angles. By leveraging these distinct field distributions, the sensor can detect analytes more effectively, which improves both the sensitivity and specificity of the detection process.

**Figure 3:**
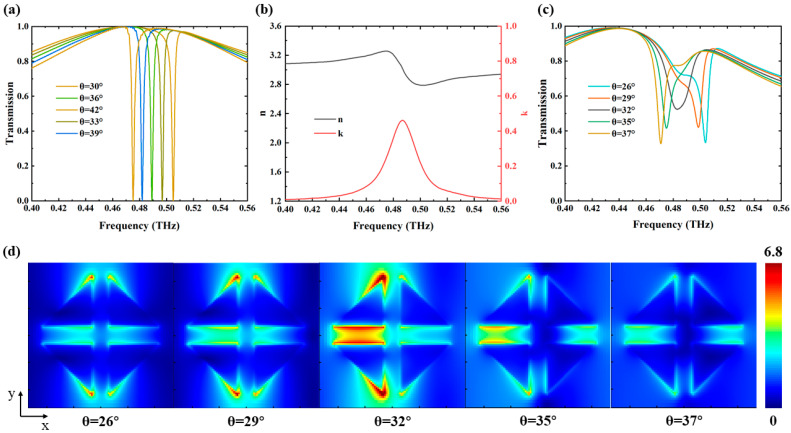
(**a**) Transmission spectra at different incident angles without any analyte; (**b**) the experimentally measured refractive index (n) and extinction coefficient (k) of cinnamoylglycine across the relevant frequency range; (**c**) transmission spectra at different incident angles with a 1 μm thick layer of analyte; (**d**) the electric field distribution measured at the substrate surface in the x–y plane at 0.487 THz for specific incident angles, corresponding to the transmission spectra shown in (**c**), respectively.

To investigate the combined spectral response and the relevancy between resonance dip and the incident angle, various angles were selected for analysis. Figure 3 illustrates that each incident angle corresponds to a distinct narrowband unity transmission, with the resonance frequency progressively decreasing as the incident angle increases. This frequency shift results from the alteration of the structure’s symmetry due to the change in the incident angle. As this asymmetry intensifies, the frequency shift becomes more observable. Furthermore, modifying the parameters of the tetramer leads to a shift in the resonance frequency of the MD [49]. These influencing factors together create a comprehensive spectral cluster within the transmission spectrum, covering a broad frequency range that aligns closely with the absorption frequency of the analyte. This relevancy is crucial for enhancing the precision of sample identification, as it ensures that the spectral response could precisely mirror the unique absorption characteristics of the analyte.

When the THz wave interacts directly with the metasurface in the absence of analyte, as shown in Figure 3a, both the minimum transmission value and the linewidth of the transmission spectra remain constant. In Figure 3b, the experimentally measured complex refractive index of cinnamoylglycine is presented, with data extracted using Fresnel formulas [51]. The real part, n, signifies the refractive index, whereas the imaginary part, k, denotes the extinction coefficient. This measurement indicates that the fingerprint absorption peak for cinnamoylglycine is located at 0.487 THz.

The fingerprint detection capability of the metasurface is evaluated by observing the transmission envelope curve with a 1 μm thick cinnamoylglycine layer. Figure 3c displays a unique envelope that peaks at 0.487 THz at an incident angle of 32°, matching the extinction coefficient curve for cinnamoylglycine. This phenomenon is explained by the electric field distributions at specific angles, shown in Figure 3d. At 0.487 THz and an incident angle of 34°, a notable electric field enhancement is observed, with pronounced concentration in the central region of the structure. This enhancement and localization increase the likelihood of wave–matter interactions near the resonant frequency for the analyte on the metamaterial. In contrast, when the incident angle is reduced to 25° or increased to 42°, the electric field strength diminishes.

The sensing sensitivity (*S*) of the designed metasurface is evaluated based on S=∆f/σ. As is shown in Figure 3c, the resonant frequency at 37° is 0.471 THz, resulting in a frequency shift ∆f of 0.0158 THz. With the 1 μm thick cinnamoylglycine layer, the surface concentration is calculated as σ=ρ×h, where ρ represents the volume density of cinnamoylglycine, noted as $1.23\;g/{cm}^{3}$. Accordingly, $\sigma =123\;\mu g/{cm}^{2}$. Figure of merit (*FoM*) is used to assess the comprehensive performance of the sensor, which is defined as $FoM=S/FWHM=S\times Q/f_{0}$ [52]. Consequently, *FoM* is obtained as 609.

**Figure 4:**
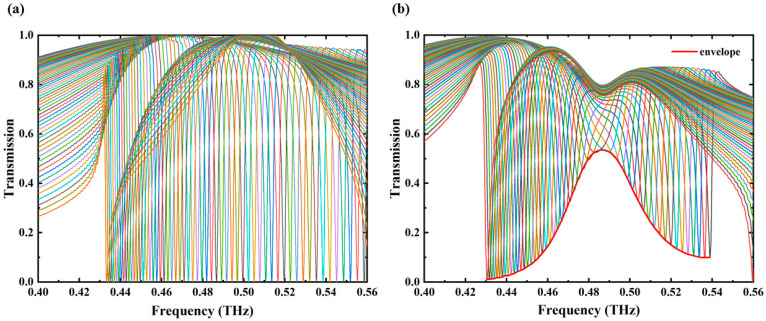
(**a**) Comprehensive transmission spectra without any analyte, with the incident angle ranging from 13° to 70°. Specifically, the rightmost line represents the transmission curve for an angle of 13°, while the leftmost line corresponds to 70°; (**b**) comprehensive transmission spectra with 1 μm thick cinnamoylglycine, with the incident angle ranging from 13° to 62°. The corresponding envelope curve has been plotted by red line in the figure.

Figure 4 presents the comprehensive angle-scanning transmission spectra of the metasurface, demonstrating that this strategy effectively distinguishes cinnamoylglycine from other substances due to its distinct absorption resonances. In Figure 4a, the transmission spectra show stable frequency shifts across various incident angles ranging from 13° to 70° in the absence of any analyte, indicating a consistent performance of the metasurface. Conversely, Figure 4b presents the transmission spectra with a 1 μm thick cinnamoylglycine layer on the metasurface. The maximum transmission rate for each incident angle was extracted, and the results were fitted into an envelope curve, plotted as the red line in Figure 4b. The envelope curve peaks at 0.487 THz, which reaches approximately 56.85%, aligning with the characteristic fingerprint spectrum of cinnamoylglycine. The observed phenomenon is attributed to the optical loss of cinnamoylglycine, indicating that the angle-scanning strategy enhances detection sensitivity and accuracy by capturing comprehensive transmission envelopes, thereby facilitating precise analyte identification.

**Figure 5:**
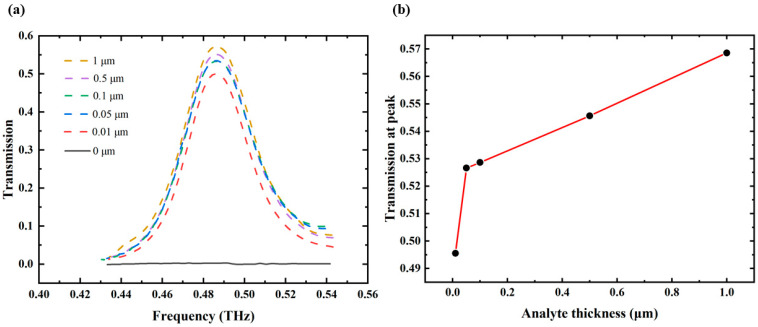
(**a**) Transmission envelope curves for analytes of varying thicknesses; (**b**) the relationship between the thickness of the analyte and the transmission at 0.487 THz.

Figure 5a depicts the transmission envelope curves for cinnamoylglycine across a series of layer thicknesses, spanning a frequency range from 0.40 THz to 0.56 THz. Notably, the transmission peak increases from 49.55% to 56.85% at 0.487 THz as the analyte layer thickness increases from 0.01 μm to 1 μm, whereas the transmission envelope remains approximately at zero in the absence of analyte. The detection limit is determined by σ=ρ×h, among which the volume density of analyte is $\rho =1.23g/{cm}^{3}$, and the minimum thickness of analyte layer is h=0.01 μm. Consequently, the detection limit is $1.23\;\mu g/{cm}^{2}$. It can be observed in Figure 5b that the transmission at 0.487 THz exhibits a linear relationship with the analyte thickness when the thickness exceeds 0.01 μm. The fitted equation is y=0.044x+0.5242, where y represents the transmission and x denotes the analyte thickness. The correlation coefficient of approximately 0.99 indicates a strong linear dependence of the fitting line, suggesting that the thickness of cinnamoylglycine can be predicted from its transmission at 0.487 THz.

## 4. Conclusions

In conclusion, this study presents a novel all-dielectric metasurface-based THz sensor for the sensitive detection of cinnamoylglycine, an emerging urinary biomarker for GDM. The proposed sensor, utilizing lithium tantalate triangular prism tetramers on a quartz substrate, achieves a high Q-factor of 231, significantly enhancing molecular fingerprint detection. The angle-scanning strategy enables broad spectral coverage and captures comprehensive transmission envelope curves that precisely align with specific analyte absorption frequencies. Simulation results indicate a detection limit as low as $1.23\;\mu g\cdot {cm}^{-2}$, demonstrating the sensor’s exceptional sensitivity and specificity. Considering the severe health risks associated with GDM, including preeclampsia and preterm birth, this approach offers a rapid, convenient, and accurate alternative to traditional diagnostic methods, with potential applications in detecting other biomarkers and monitoring various diseases. It meets the critical need for non-invasive and efficient GDM monitoring, facilitating regular monitoring and timely intervention. The successful identification of cinnamoylglycine underscores the robust performance of the metasurface in practical sensing applications, establishing a solid foundation for future research and advancements in THz spectroscopy and biomedical diagnostics.

## Data Availability

The data underlying the results presented in this paper may be obtained from the authors upon reasonable request.
